# Correspondence regarding ‘Assefa Y, et al., BMC Health Services Research. 2011; 11 (1):81 and 2014; 14(1):45’: The Positive-Deviance approach for translating evidence into practice to improve patient retention in HIV care

**DOI:** 10.1186/s12913-018-3018-9

**Published:** 2018-03-21

**Authors:** Yibeltal Assefa, Peter S. Hill, Helmut Kloos, Gorik Ooms, Wim Van Damme

**Affiliations:** 1 School of Public Health, the University of Queensland, Brisbane, Australia; 2 Ethiopian Public Health Institute, Addis Ababa, Ethiopia; 3 Department of Epidemiology and Biostatistics, University of California, San Francisco, USA; 4 Department of Global Health and Development, Faculty of Public Health and Policy, London School of Hygiene & Tropical Medicine, London, UK; 5 Department of Public Health, Institute of Tropical Medicine, Antwerp, Belgium

**Keywords:** Antiretroviral treatment, Retention in care, Positive deviance, Translating evidence into practice

## Abstract

The purpose of this correspondence is to describe how the positive-deviance approach can be used to translate evidence into practice, based on successive studies conducted in Ethiopia. In earlier studies, it was identified that retention in antiretroviral treatment care was variable across health facilities; and, seeking compliance across facilities, a framework was developed based on the practices of those positive-deviant health facilities, where performance was noted to be markedly better. It was found that the positive deviance approach was effective in facilitating the transfer of innovative practices (using different mechanisms) from positive-deviant health facilities to negative-deviant health facilities. As a result, the variability in retention in care across health facilities narrowed over time, increasing from 83 to 96% in 2007/8 to 95-97% in 2013/14. In conclusion, the positive-deviance approach is a valuable tool to translate evidence into practice, spread good practices, and help achieving universal health coverage.

## Background

Retention in care in the Ethiopian antiretroviral treatment (ART) program, based on a study conducted in 2009 in randomly selected 55 health facilities from all parts of the country, was found to be variable, ranging from 51to 85%, across these health facilities [1]. Analysis of nine health facilities (selected from the 55 health facilities) in 2009 and 2011 showed that retention in care was still variable; and, health facilities with higher retention in care implemented a more comprehensive package of interventions which were not available in health facilities with lower retention in care. In response to this finding, a framework to improve retention in care was developed by applying the positive-deviance (PD) approach [2].

The PD approach is a behavioral change approach based on the observation that in any context, certain individuals or organizations, confronting challenges and resource deprivations similar to those of their peers, will employ uncommon but successful behaviors or interventions which enable them to tackle their problems [3, 4]. The PD approach assumes that innovative solutions to common challenges may be identified through the study of individuals or organizations (referred to as ‘positive deviants’) which demonstrate outlying behaviors. Such behaviors are thought to be acceptable and sustainable because they are already practiced by individuals or organizations that are facing similar challenges [3, 4].

The purpose of this correspondence is to systematically describe the PD approach, focusing mainly on the outcomes of its final phase: spreading good practices of positive deviant health facilities to other health facilities, with the intention to improve patient retention in care in Ethiopia [1, 2].

### Positive-deviance approach to translate evidence into practice

The PD approach was applied in two phases, step-by-step, using data in the ART program in Ethiopia between 2005/6 and 2013/14. It was employed to achieve two objectives in two phases: (1) to develop a framework to improve retention in care in the ART program (phase one) [2], and (2) to monitor and evaluate the dissemination and effectiveness of the framework in improving retention in care (phase two).

Phase one (using data in 2005/6-2008/9) was conducted in 2009 and 2011, and had four steps: defining the challenge and desired outcome, determining the positive-deviant health facilities (based on data in 2008/9), discovering uncommon but successful interventions, and developing a framework [1, 2]. In this phase, retention in care was found to be variable, but those health facilities with higher and improving retention (positive deviants) were found to implement a comprehensive package of interventions, which were either poorly implemented or did not exist in health facilities with lower retention in care (negative deviants) [2]. Accordingly, a framework for retention in care was developed and the findings from this phase were published in BMC Health Services Research [2].

A follow on (phase two) study (using data in 2009/10-2013/14) was subsequently conducted in 2014 to monitor and evaluate the dissemination and effectiveness of the framework in three negative-deviant health facilities, comparing them to two positive-deviant health facilities. This was conducted in three steps: developing an action plan (for implementing the interventions in the framework) with health facilities which had lower retention in care in phase one, executing the action plan, and monitoring and evaluating the effectiveness of the framework for retention in care.

A debriefing and review meeting was conducted with representatives from these health facilities, program managers, and implementing partners on the interventions to improve retention in care. An action plan to improve retention in care was developed, indicators were selected for each activity that was used to monitor and evaluate the implementation of the action plan to improve retention in care. Health facilities were assisted in mobilizing the necessary resources (both financial and technical) from their local government and implementing partners to execute their plans of action. The implementation of the framework and levels of retention in care were regularly monitored and reviewed.

### Improved retention in care in negative-deviant health facilities

**Fig. 1:**
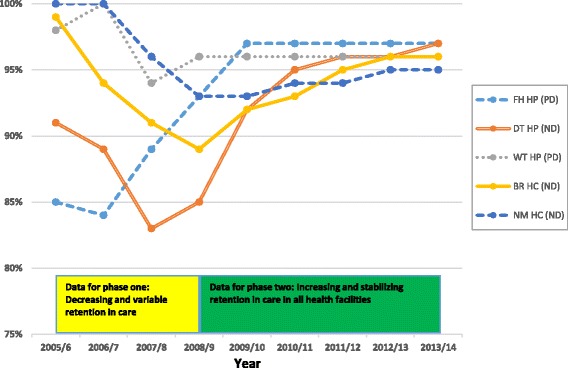
Trend in retention in care in three negative-deviant and two positive-deviant health facilities in Ethiopia, 2005/6-2013/14

On evaluation, it was found that the health facilities were able to introduce and implement the interventions through a variety of management practices (including review meetings, supportive supervision activities, and exchange of best practices with other health facilities). The levels of retention in care were improving, and the gap in level of retention in care among health facilities narrowed over time: from between 83 and 96% in 2007/8 to between 95 and 97% in 2013/14 (Fig. 1). The PD approach was thus found to be a valuable tool for improving retention in care in the Ethiopian ART program.

### Positive-deviance approach can be a valuable tool towards universal health coverage

The Ethiopian ART program benefited from the PD approach to develop strategies to improve retention in care when the program faced a real challenge of retaining patients in care [2, 5]. This was especially possible due to the variability of retention in care across health facilities in the country [1, 2]. The PD approach is appropriate where there is variability in performance across organizations, with some achieving improved and consistently high performance while others are failing to improve their performance. The success of the approach depends on: (1) the ability to identify positive deviants which use uncommon strategies that help to tackle common problems [3, 6], (2) the willingness of positive deviants to share their effective approaches with negative deviants [6, 7], (3) the perceived need for improvement by negative deviants [3, 7], and (4) the application and corroboration of the framework (developed based on the experience of positive deviants) in negative deviants [8, 9].

The PD approach basically combines a mix of methods (both qualitative and quantitative), and integrates some of the strengths of other approaches [10, 11]. The PD approach not only studies processes but also analyses contexts in which strategies are implemented. In this respect, it has similarities with realist evaluation [12]. A recent systematic review of the methods used in the PD approach highlights the approach’s flexibility, relevance to a range of quality improvement issues, and use in identifying practical and sustainable solutions in health care [13].

However, the PD approach is not without limitation: the apparent limited generalizability of findings from one context to other contexts. This may hinder the implementation and effectiveness of the recommendations in wider contexts. Moreover, finding ‘transferable practices’ that can improve performance may not always be possible. This is especially true in well-established programs such as immunization where the entire toolkit to reach high coverage is well established compared to newer programs, such as ART, in which innovative practices are still being discovered and can be transferred to others. This study also had a limitation in that it did not include a comparison group of negative-deviant health facilities (which were identified in phase one) [2] which did not implement the interventions in the framework.

In conclusion, the PD approach was recognized to be helpful for identifying innovative solutions, based on the variability of performance among health facilities, and facilitated the translation of evidence-based successful strategies, identified from the positive deviants, into practice in the negative-deviants. This also enabled the negative-deviant health facilities to improve their performance. We thus believe that the PD approach can be a valuable tool for researchers, policy makers, program managers, funders, and global health initiatives in their efforts to improve performance of health services and programs towards universal health coverage.

## Abbreviations

ART: Antiretroviral treatment
HC: Health Center
HP: Hospital
ND: Negative deviance
PD: Positive deviance
